# Mycoplasma pneumoniae Infection With Cavitated Lung Lesions: A Case Report

**DOI:** 10.7759/cureus.31572

**Published:** 2022-11-16

**Authors:** Ana Luísa Campos, Filipa Madalena F Gonçalves, Magda Costa, Glória Alves, Jorge Cotter

**Affiliations:** 1 Internal Medicine, Hospital da Senhora da Oliveira, Guimarães, PRT

**Keywords:** cavitated lung lesions, azithromycin, community-acquired pneumonia, mycoplasma pneumoniae, atypical pneumonia

## Abstract

*Mycoplasma pneumoniae *(MP) is a common etiologic agent involved in community-acquired atypical bacterial pneumonia. In severe cases, *M. pneumoniae* can cause cavitated lung lesions. We describe the case of a 55-year-old male seen at the emergency department with complaints of cough, fever, dyspnea, pleuritic chest pain, nausea, anorexia, asthenia, and night sweats. Cavitated lesions in the upper lobes of both lungs were documented on thoracic computed tomography (CT). An extensive investigation ruled out several infectious and non-infectious causes. The only positive result was a high immunoglobulin M (IgM) titer for *M. pneumoniae*. The patient was treated with azithromycin and exhibited rapid clinical improvement. Three months later, a repeat thoracic computed tomography showed the resolution of the cavitated lesions. In this rare case of *M. pneumoniae* pneumonia with cavitated lesions, early identification of the etiologic agent and prompt antibiotic therapy led to the resolution of the cavitated lung lesions.

## Introduction

*Mycoplasma pneumoniae* (MP) is a common bacterial cause of community-acquired pneumonia. However, because clinical and radiological findings are variable and nonspecific and the few diagnostic methods available offer variable levels of precision, diagnosing MP pneumonia presents a difficult challenge for clinicians [1]. The most common findings on chest radiographs and computed tomography (CT) scans of patients with MP pneumonia are unilateral or bilateral areas of airspace consolidation or ground glass opacity [2]. Though rare, severe forms of MP infection may cause cavitated lesions [1,3,4]. The aim of this article is to describe a rare case of bilateral cavitated lung lesions caused by MP pneumonia.

## Case presentation

A 55-year-old male came to the emergency department after one week of daily fever (maximum temperature of 39°C) that responded to antipyretics, along with night sweats, asthenia, anorexia, nausea, dyspnea after medium exertion, pleuritic chest pain, and initially dry then productive cough with mucopurulent secretions. The patient had a history of arterial hypertension, dyslipidemia, and ischemic heart disease with acute myocardial infarction 10 years before. He was an active tobacco smoker with a 40-pack-year history. The patient also had a history of chronic alcoholism but had been abstinent for several years. He was on daily medications including an acetylsalicylic acid, a statin, an angiotensin-converting enzyme inhibitor, a beta-blocker, and a proton pump inhibitor. There was no history of immunosuppressive medication.

Upon admission to the emergency department, the patient was normotensive and normocardic with peripheral oxygen saturation (SpO_2_) of 96% in room air and a tympanic temperature of 37.9°C. On physical examination, there was an emaciated appearance, pallor of the skin and mucous membranes, hoarse voice, and a reddish, non-pruritic, macular rash on the supraclavicular region. The examination of the oral cavity and oropharynx was normal. No other abnormalities were noted, including on pulmonary auscultation.

As shown in Table 1, blood analysis revealed normocytic/normochromic anemia, leukocytosis with neutrophilia, mild thrombocytosis, moderate elevation of liver enzymes, slight prolongation of prothrombin time, and marked elevation of C-reactive protein. The electrocardiogram revealed no changes, and the high-sensitivity troponin I was normal. No respiratory failure or acid-base disturbances were documented in arterial blood gas analysis. The polymerase chain reaction (PCR) test for the detection of SARS-CoV-2 virus was negative. Urine tests for *Legionella pneumophila* and *Streptococcus pneumoniae* antigens were negative.

**Table 1:** Blood analysis results

	Test result	Reference value
Hemoglobin	11.2	14.0-18.0 g/dL
Mean corpuscular volume	89.4	83-103 fL
Mean corpuscular hemoglobin	30.4	28-34 pg
Mean corpuscular hemoglobin concentration	33.9	32.0-36.0 g/dL
White blood cells	16	4.8-10.8 (×10^3^/µL)
Neutrophils	12.6	1.8-7.7 (×10^3^/µL)
Eosinophils	0.0	0.00-0.49 (×10^3^/µL)
Basophils	0.0	0.0-0.1 (×10^3^/µL)
Lymphocytes	1.8	1.0-4.8 (×10^3^/µL)
Monocytes	1.4	0.12-0.80 (×10^3^/µL)
Platelets	441	150-350 (×10^3^/µL)
C-reactive protein	223.7	<3.0 mg/L
Urea	37	15-39 mg/dL
Creatinine	0.88	0.70-1.30 mg/dL
Sodium	137	135-146 mEq/L
Potassium	4.45	3.5-5.1 mEq/L
Total bilirubin	0.52	0.3-1.2 mg/dL
Direct bilirubin	0.33	0.0-0.3 mg/dL
Aspartate aminotransferase	115	12-40 UI/L
Alanine aminotransferase	159	7-40 UI/L
Gamma-glutamyl transferase	439	0-73 UI/L
Alkaline phosphatase	349	46-116 UI/L
Lactate dehydrogenase	267	120-246 UI/L
Prothrombin time	16	8.4-14.4 seconds
Activated partial thromboplastin time	27	20.9-34.9 seconds

**Figure 1:**
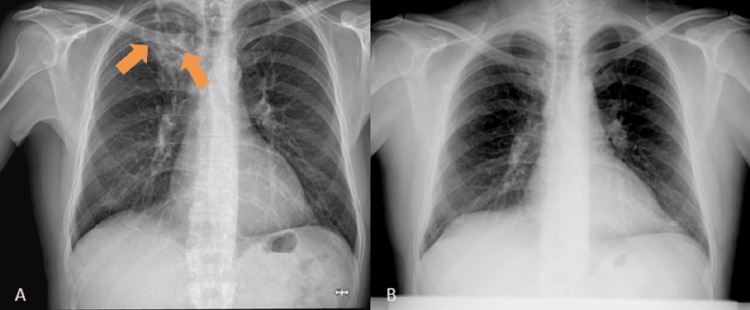
Images of the chest radiographs (A) Chest radiography taken on the day of admission to the emergency department, showing heterogeneous rounded hypertransparency of important dimensions at the apex of the right lung (orange arrows). (B) Chest radiography performed 10 years before, showing no abnormalities

A chest radiography was performed, documenting heterogeneous hypertransparency with rounded contours and hypotransparent areas at the apex of the right lung. In comparison to a chest radiography taken 10 years earlier, the hypertransparency was de novo (Figure 1).

**Figure 2:**
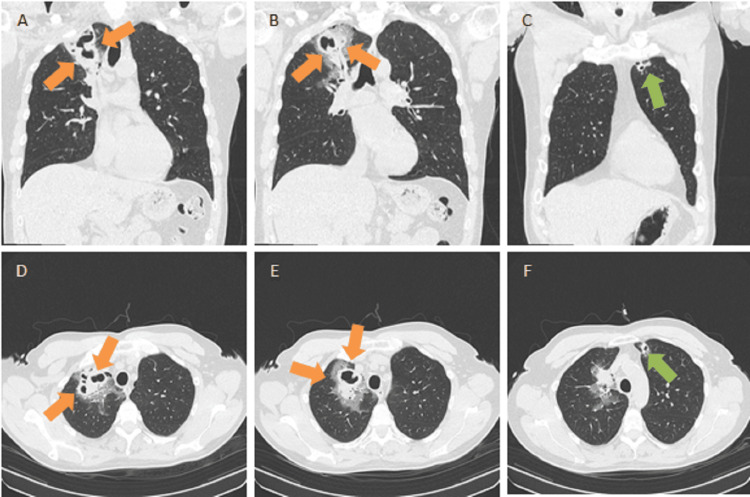
Images of the thoracic computed tomography performed at admission (A and B) Coronal views showing partially cavitated lesions in the right upper lobe (orange arrows). (C) Coronal view showing cavitated lesion in the left upper lobe (green arrow). (D and E) Axial views showing partially cavitated lesions in the right upper lobe (orange arrows). (F) Axial view showing cavitated lesion in the left upper lobe (green arrow)

To better characterize the alterations found on the radiography, a thoracic CT scan was performed. The results showed two partially cavitated areas of parenchymal densification in the right upper lobe, approximately 4.5 cm on the longest axis with adjacent ground glass opacity, and a cavitated lesion 1.5 cm on its longest axis in the left upper lobe (Figure 2). An abdominal CT scan performed at the same time showed no hepatic or other alterations.

The patient was admitted to an airborne infection isolation room for suspicion of pulmonary tuberculosis and started on ceftriaxone and azithromycin antibiotics for the empirical treatment of bilateral community-acquired pneumonia. The human immunodeficiency virus (HIV) serological test was negative. Immunoglobulins A, M, and G (IgA, IgM, and IgG) were within normal ranges. Serological tests for Epstein-Barr virus, herpes simplex virus 1 and 2, and cytomegalovirus were negative. The patient was immune to toxoplasmosis and rubella. There was no serological evidence of *Chlamydia pneumophila* or *Legionella pneumophila* infection. However, the MP serological test revealed a positive IgM titer and a negative IgG titer, which is compatible with acute infection (IgM 15 and IgG 1.9, reference value >10).

On day 4 of hospitalization, the patient was apyretic. On day 5, he reported significant symptomatic improvement. After four sputum samples tested negative for mycobacteria on Ziehl-Neelsen stain, isolation was suspended, and we stopped treatment with ceftriaxone but maintained the azithromycin. On day 11, the patient had reduced frequency of coughing, reduced sputum with mucous characteristics, and a rash on the trunk and dysphonia completely resolved. Leukocytosis with neutrophilia and thrombocytosis also resolved. C-reactive protein dropped dramatically to 16 mg/L, and liver enzymes and prothrombin time returned to normal values. No bacteria were isolated in the sputum culture examinations, and no mycobacteria were found on Ziehl-Neelsen stain (total of five samples). Blood cultures were also negative.

**Figure 3:**
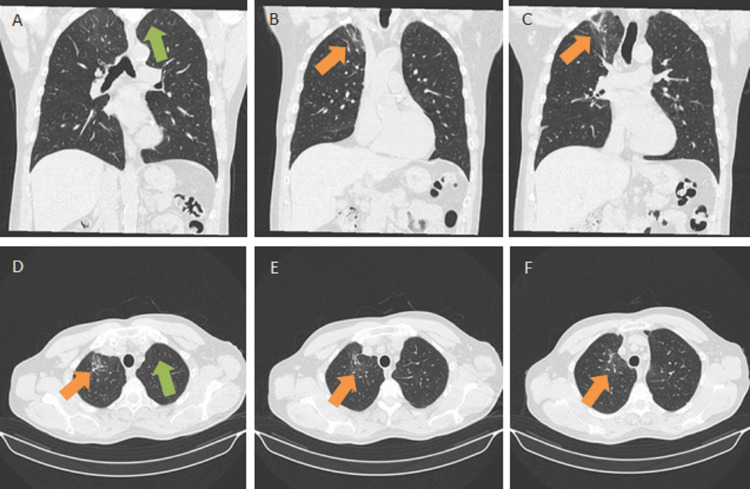
Images of the thoracic computed tomography performed three months after hospitalization (A) Coronal view showing complete resolution of cavitated lesion in the left upper lobe (green arrow). (B and C) Coronal views showing residual fibro-retractable alterations in the right upper lobe (orange arrows). (D) Axial view showing complete resolution of cavitated lesion in the left upper lobe (green arrow). (D, E, and F) Axial views showing residual fibro-retractable alterations in the right upper lobe (orange arrows)

The patient was discharged on day 11 of hospitalization after 10 days of treatment with azithromycin. At the time of discharge, *Mycobacterium tuberculosis* DNA tests were still in progress for the five sputum samples. The patient was oriented for an internal medicine consultation. Two weeks later, a fiber-optic bronchoscopy and bronchoalveolar lavage of the apical segment of the right upper lobe revealed no malignant cells and no bacteria in the cytological analysis or cultures. Ziehl-Neelsen staining of bronchial aspirate was negative for *M. tuberculosis*, as was the DNA test of sputum samples collected during hospitalization. A thoracic CT scan was performed three months after hospital admission. As shown in Figure 3, the CT scan revealed complete resolution of the cavitated lesion in the left upper lobe and almost complete resolution of the lesion of the right upper lobe (remaining in these topography-discrete fibro-retractable alterations without cavitations).

The patient’s rapid improvement after treatment with azithromycin, the positivity for IgM (with negative IgG) of MP, and the lack of other infectious or non-infectious causes upon complementary examinations led to a definitive diagnosis of a rare case of cavitated lung lesions caused by MP. The resolution of the lesions at three months after infection corroborated the diagnosis. Currently, at one year after hospital admission, the patient is completely asymptomatic, with no alterations in the physical examination and no significant changes in the most recent analytical study.

## Discussion

MP is a common agent of community-acquired lower respiratory tract infections, particularly among adults, the immunocompromised, smokers, and those with lung disease [3]. MP also is a causative agent of atypical pneumonia in which systemic rather than respiratory manifestations predominate; there are bilateral patchy infiltrates on chest radiographs, and sputum cultures and Gram stains are negative [5]. Each agent of atypical pneumonia has a predilection for different extrapulmonary organs and therefore a different pattern of extrapulmonary involvement. For example, in MP infections, the involvement of the upper respiratory tract can manifest as non-exudative pharyngitis and laryngitis, as well as skin involvement [6]. In this report, we describe a patient with an active tobacco-smoking habit who presented with hoarseness and a macular rash in the supraclavicular region.

MP infection is usually mild and self-limiting, with symptoms similar to an upper respiratory tract infection, such as the indolent development of headache, malaise, low-grade fever, bronchitis, cough, and, in 3%-10% of patients, pneumonia [5], as well as the involvement of the gastrointestinal tract [6]. In the acute phase of infection, a dry cough typically develops and progresses to a productive cough within three to four days [7]. Cough secondary to tracheobronchitis is the most common manifestation of MP infection. Our patient exhibited many of these symptoms. The mild thrombocytosis and hepatitis we observed also are well described in the literature [1].

Cavitated lung lesions can be either infectious or non-infectious. Infectious causes are numerous, and non-infectious causes include primary lung neoplasms, metastatic lesions and vasculitis, sarcoidosis, and other autoimmune diseases [3]. In a retrospective study analyzing 203 cases of adults admitted to a Lebanese hospital with a diagnosis of MP pneumonia over seven years, only two (~1%) had cavitated lesions [4]. In retrospect, the patient’s clinical picture was typical of MP infection. However, due to the presence of cavitated lesions, which are rarely associated with MP pneumonia [1,3,4], it was important to rule out other causes.

An extensive battery of tests ruled out viral, bacterial, and mycobacterial causes, including *M. tuberculosis*. No neoplastic cells were found, and no infectious agent was identified in the bronchoalveolar lavage or bronchial aspirate. Even without excluding all non-infectious causes, the patient’s rapid clinical and analytical improvement after starting empirical antibiotic therapy led us to believe that the cause was likely infectious.

On day 5 of hospitalization, the MP IgM result was positive. An acutely elevated IgM titer in a symptomatic patient is considered diagnostic of infection [4,6]. MP has no cell wall, so it is not visible on Gram stain [3]. Moreover, the bacterial culture for MP is time-consuming due to nutritional requirements and has low sensitivity, so this diagnostic technique is used for research purposes and not in clinical practice. For these reasons, we did not identify MP in routine cultural examinations. Serology remains the most common and relevant technique for diagnosing MP infection [7].

As MP has no cell wall, penicillin and cephalosporins are not effective in treatment [5]. The choice of antibiotic is limited to those that act on the ribosome of the bacteria, interfering with protein synthesis, as is the case with macrolides. Azithromycin is the macrolide of choice as it is better tolerated and has a long half-life, allowing for shorter courses of treatment. In addition to their bacteriostatic effect, macrolides have an anti-inflammatory effect, leading to the resolution of infection via a synergistic mechanism [1]. Our patient completed 10 days of azithromycin with rapid improvement. The optimal duration of treatment is unclear, but 10-14 days are generally recommended [7]. When the resolution of cavitated lesions was documented on a chest CT performed three months after treatment, we confirmed a rare case of cavitated lung lesions caused by MP.

## Conclusions

MP is a relatively common cause of community-acquired pneumonia, but its association with cavitated lung lesions is rare, especially in immunocompetent patients. In this article, we describe a rare case of lung cavitated lesions in an immunocompetent patient with community-acquired pneumonia by MP. The timely identification of the etiologic agent via serology facilitated early and appropriate treatment, leading to rapid clinical improvement and the resolution of the cavitated lesions.
